# Tetra­ethyl­ammonium toluene-4-sulfon­ate

**DOI:** 10.1107/S1600536813002961

**Published:** 2013-02-02

**Authors:** Diana Malgorzata Brus, Justyna Czyrko, Krzysztof Brzezinski

**Affiliations:** a Institute of Chemistry, University of Bialystok, Hurtowa 1, 15-399 Bialystok, Poland

## Abstract

There are two tetra­ethyl­ammonium cations and two toluene-4-sulfate anions in the asymmetric unit of the title salt, C_8_H_20_N^+^·C_7_H_7_O_3_S^−^. One of the anions is disordered over two positions, with refined occupancies of 0.447 (3) and 0.553 (3). In the crystal, the cations and anions are linked by C—H⋯O hydrogen bonds, forming ribbons along [10-1]. The ribbons are linked *via* C—H⋯O hydrogen bonds, forming a two-dimensional network lying parallel to (10-1).

## Related literature

For the preparation of tetra­ethyl­ammonium toluene-4-sulfonate from ethyl 4-toluene­sulfonate and triethyl­amine, see: Baizer (1964 ▶). For its application as a phase-transfer catalyst, see: Cerveau *et al.* (2002 ▶) or as the supporting electrolyte, see: Adachi *et al.* (1979 ▶); Wynne & Street (1985 ▶); Yoshida *et al.* (1986 ▶); Wong & Moeller (1993 ▶); Ben *et al.* (2011 ▶).
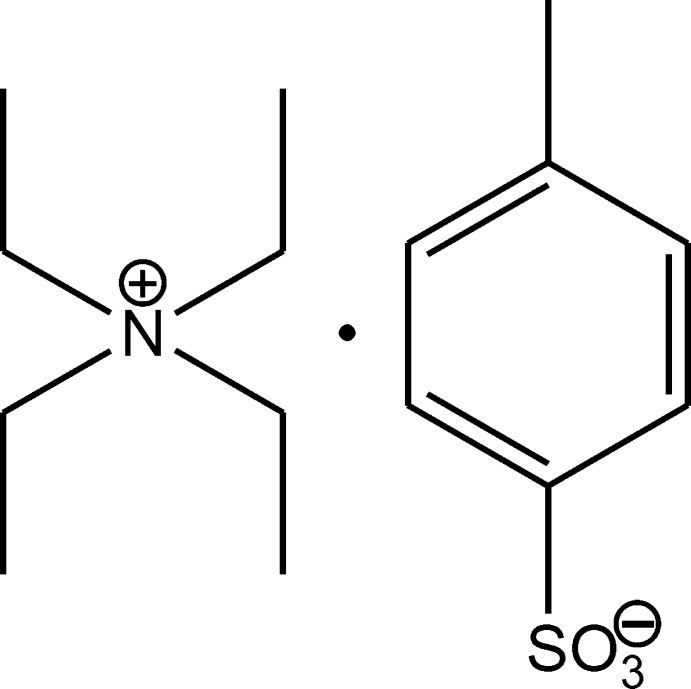


## Experimental

### 

#### Crystal data

C_8_H_20_N^+^·C_7_H_7_O_3_S^−^
*M* *_r_* = 301.21
Monoclinic, 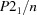

*a* = 16.8771 (3) Å
*b* = 7.53713 (16) Å
*c* = 26.2404 (6) Å
β = 97.2938 (18)°
*V* = 3310.90 (12) Å^3^
*Z* = 8
Mo *K*α radiation
μ = 0.20 mm^−1^
*T* = 100 K
0.8 × 0.6 × 0.3 mm

#### Data collection

Agilent SuperNova (Dual, Cu at zero, Atlas) diffractometer
Absorption correction: multi-scan (*CrysAlis PRO*; Agilent, 2011 ▶) *T*_min_ = 0.771, *T*_max_ = 1.000
6276 measured reflections
6276 independent reflections
5477 reflections with *I* > 2σ(*I*)
*R*_int_ = 0.050

#### Refinement

*R*[*F* ^2^ > 2σ(*F* ^2^)] = 0.076
*wR*(*F* ^2^) = 0.163
*S* = 1.19
6276 reflections
406 parameters
82 restraints
H-atom parameters constrained
Δρ_max_ = 0.46 e Å^−3^
Δρ_min_ = −0.50 e Å^−3^

### 

Data collection: *CrysAlis PRO* (Agilent, 2011 ▶); cell refinement: *CrysAlis PRO*; data reduction: *CrysAlis PRO*; program(s) used to solve structure: *SHELXD* (Sheldrick, 2008 ▶); program(s) used to refine structure: *SHELXL97* (Sheldrick, 2008 ▶); molecular graphics: *OLEX2* (Dolomanov *et al.*, 2009 ▶); software used to prepare material for publication: *SHELXL97*.

## Supplementary Material

Crystal structure: contains datablock(s) global, I. DOI: 10.1107/S1600536813002961/kp2445sup1.cif

Structure factors: contains datablock(s) I. DOI: 10.1107/S1600536813002961/kp2445Isup2.hkl

Supplementary material file. DOI: 10.1107/S1600536813002961/kp2445Isup3.cml

Additional supplementary materials:  crystallographic information; 3D view; checkCIF report

## Figures and Tables

**Table 1:** Hydrogen-bond geometry (Å, °)

*D*—H⋯*A*	*D*—H	H⋯*A*	*D*⋯*A*	*D*—H⋯*A*
C6*B*—H6*B*⋯O23	0.95	2.57	3.351 (6)	140
C31—H31*B*⋯O3*B* ^i^	0.99	2.49	3.344 (4)	145
C33—H33*A*⋯O2*B*	0.99	2.47	3.354 (4)	148
C35—H35*A*⋯O22^ii^	0.99	2.42	3.228 (4)	138
C36—H36*C*⋯O3*B* ^iii^	0.98	2.58	3.544 (4)	169
C43—H43*B*⋯O22	0.99	2.44	3.269 (4)	141
C45—H45*A*⋯O2*B*	0.99	2.53	3.367 (4)	142
C47—H47*A*⋯O3*B* ^i^	0.99	2.57	3.440 (4)	147
C48—H48*B*⋯O22^iv^	0.98	2.58	3.562 (4)	175

Symmetry codes: (i) 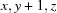; (ii) 
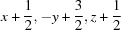
; (iii) 
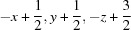
; (iv) 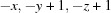.

**Fig. 1.**
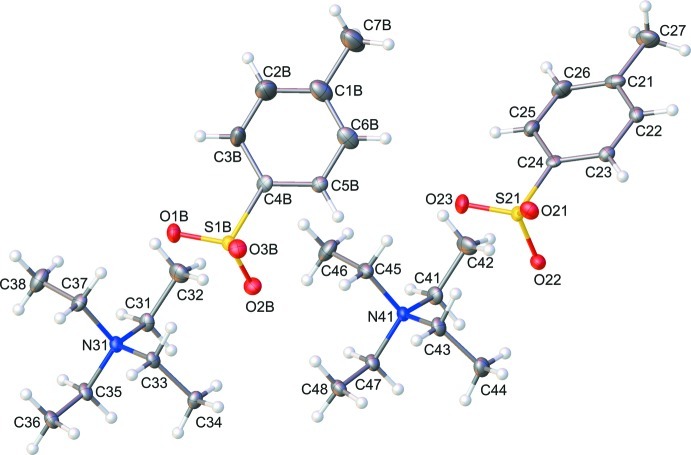
The molecular structure of the title compound. Displacement ellipsoids are drawn at the 50% probability level. For clarity, only more populated location of the disordered anion (B) is shown.

**Fig. 2.**
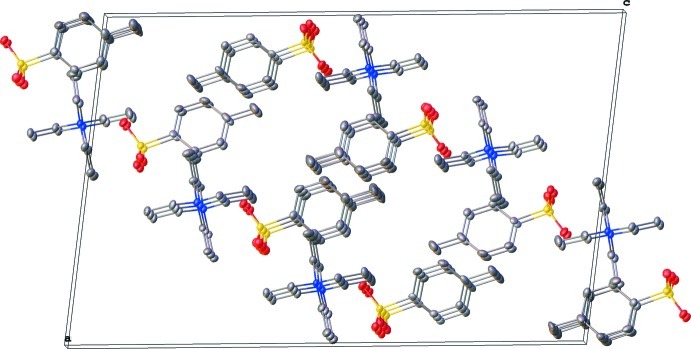
Crystal packing viewed along b direction. For clarity, hydrogen atoms are ommited.

**Fig. 3.**
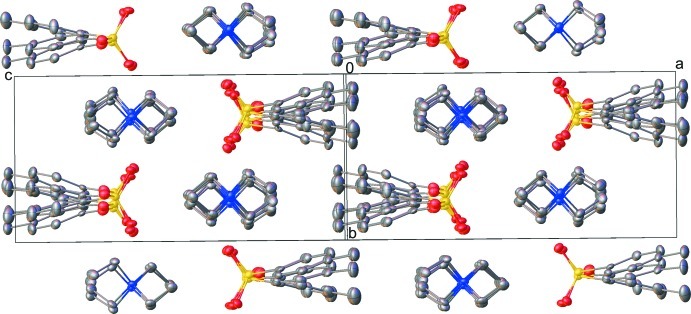
Crystal packing viewed along ac direction. For clarity, hydrogen atoms are ommited.

## supplementary crystallographic information

### Comment

Tetraethylammonium toluene-4-sulfonate is applied as the phase-transfer catalyst in the preparation of bis-silanetriols (Cerveau *et al.*, 2002). The compound is also widely used in electrochemistry as the supporting electrolyte (Wynne *et al.*, 1985; Yoshida *et al.*, 1986; Wong *et al.*, 1993; Ben *et al.*, 2011), because it could be easly removed from the reaction by the extraction with water (Adachi *et al.*, 1979).

The asymmetric unit contains two tetraethylammonium cations and two toluene-4-sulfate anions (Fig. 1). One of the toluene-4-sulfate ions is disordered and is modeled in the two locations. The occupancy of two major positions in the final model is refined to 0.447 (3) and 0.553 (3). Within the crystal lattice the columns of cations and anions are formed along *b* and *ac* directions (Figs. 2 and 3, respectively).

### Experimental

The title compound was prepared according to the procedure described by Baizer (1964). Briefly, ethyl toluene-4-sulfonate (200 g, 1.0 mole) was dissolved in 100 mL of anhydrous ethanol and triethylamine was added (101 g, 1.0 mole). The reaction mixture was stirred and heated under reflux for 6 h. The excess of triethylamine and ethanol was removed *in vacuo*. The crude product was washed several times with a dry ethylether and recrystallized from ethanol.

### Refinement

The disordered toluene-4-sulfate anion is modeled at the two locations with geometric (FLAT instruction) and dispacement parameter (SIMU instruction) restraints and with AFIX 66, EADP and EXYZ constraints. Seven reflections for which I(obs) and I(calc) differed more then 10 times SigmaW were ommited from the refinement. All H atoms were initially located in electron density difference maps. Hydrogen atoms were constrained to idealised positions with C—H distances fixed at 0.95–0.99 Å and 1.5*U*_eq_(C) for methyl hydrogen atoms and 1.2*U*_eq_(C) for others.

### Crystal data

**Table:**

C_8_H_20_N^+^·C_7_H_7_O_3_S^−^	*F*(000) = 1312
*M**_r_* = 301.21	*D*_x_ = 1.209 Mg m^−^^3^
Monoclinic, *P*2_1_/*n*	Mo *K*α radiation, λ = 0.71073 Å
Hall symbol: -P 2yn	Cell parameters from 9780 reflections
*a* = 16.8771 (3) Å	θ = 2.6–25.6°
*b* = 7.53713 (16) Å	µ = 0.20 mm^−^^1^
*c* = 26.2404 (6) Å	*T* = 100 K
β = 97.2938 (18)°	Plate, colourless
*V* = 3310.90 (12) Å^3^	0.8 × 0.6 × 0.3 mm
*Z* = 8	

### Data collection

**Table:**

Agilent SuperNova (Dual, Cu at zero, Atlas) diffractometer	6276 independent reflections
Radiation source: SuperNova (Mo) X-ray Source	5477 reflections with *I* > 2σ(*I*)
Mirror monochromator	*R*_int_ = 0.050
Detector resolution: 10.4052 pixels mm^-1^	θ_max_ = 25.7°, θ_min_ = 2.7°
ω scans	*h* = −20→20
Absorption correction: multi-scan (*CrysAlis PRO*; Agilent, 2011)	*k* = 0→9
*T*_min_ = 0.771, *T*_max_ = 1.000	*l* = 0→31
6276 measured reflections	

### Refinement

**Table:**

Refinement on *F*^2^	Primary atom site location: structure-invariant direct methods
Least-squares matrix: full	Secondary atom site location: difference Fourier map
*R*[*F*^2^ > 2σ(*F*^2^)] = 0.076	Hydrogen site location: inferred from neighbouring sites
*wR*(*F*^2^) = 0.163	H-atom parameters constrained
*S* = 1.19	*w* = 1/[σ^2^(*F*_o_^2^) + (0.0161*P*)^2^ + 13.1727*P*] where *P* = (*F*_o_^2^ + 2*F*_c_^2^)/3
6276 reflections	(Δ/σ)_max_ < 0.001
406 parameters	Δρ_max_ = 0.46 e Å^−^^3^
82 restraints	Δρ_min_ = −0.50 e Å^−^^3^

### Special details

**Table:**

Geometry. All e.s.d.'s (except the e.s.d. in the dihedral angle between two l.s. planes) are estimated using the full covariance matrix. The cell e.s.d.'s are taken into account individually in the estimation of e.s.d.'s in distances, angles and torsion angles; correlations between e.s.d.'s in cell parameters are only used when they are defined by crystal symmetry. An approximate (isotropic) treatment of cell e.s.d.'s is used for estimating e.s.d.'s involving l.s. planes.
Refinement. Refinement of *F*^2^ against ALL reflections. The weighted *R*-factor *wR* and goodness of fit *S* are based on *F*^2^, conventional *R*-factors *R* are based on *F*, with *F* set to zero for negative *F*^2^. The threshold expression of *F*^2^ > σ(*F*^2^) is used only for calculating *R*-factors(gt) *etc*. and is not relevant to the choice of reflections for refinement. *R*-factors based on *F*^2^ are statistically about twice as large as those based on *F*, and *R*- factors based on ALL data will be even larger.

### Fractional atomic coordinates and isotropic or equivalent isotropic displacement parameters (Å^2^)

**Table:**

	*x*	*y*	*z*	*U*_iso_*/*U*_eq_	Occ. (<1)
C1A	0.4305 (4)	0.3064 (10)	0.48865 (18)	0.0178 (18)	0.447 (3)
C2A	0.4856 (3)	0.3393 (9)	0.5316 (2)	0.0227 (19)	0.447 (3)
H2A	0.5390	0.3704	0.5276	0.027*	0.447 (3)
C3A	0.4626 (4)	0.3266 (9)	0.58052 (18)	0.0182 (18)	0.447 (3)
H3A	0.5002	0.3491	0.6099	0.022*	0.447 (3)
C4A	0.3845 (4)	0.2811 (15)	0.5864 (3)	0.019 (4)	0.447 (3)
C5A	0.3293 (3)	0.2482 (18)	0.5434 (4)	0.0163 (8)	0.447 (3)
H5A	0.2760	0.2171	0.5475	0.020*	0.447 (3)
C6A	0.3524 (3)	0.2609 (15)	0.4946 (3)	0.017 (4)	0.447 (3)
H6A	0.3147	0.2384	0.4652	0.021*	0.447 (3)
C7A	0.4550 (6)	0.3225 (15)	0.4349 (4)	0.032 (2)	0.447 (3)
H7AA	0.4883	0.2208	0.4281	0.048*	0.447 (3)
H7AB	0.4853	0.4324	0.4324	0.048*	0.447 (3)
H7AC	0.4071	0.3248	0.4095	0.048*	0.447 (3)
O1A	0.41953 (15)	0.2857 (4)	0.68544 (10)	0.0208 (6)	0.447 (3)
O2A	0.29813 (16)	0.4298 (3)	0.64542 (10)	0.0208 (6)	0.447 (3)
O3A	0.30363 (16)	0.1087 (3)	0.65189 (10)	0.0208 (6)	0.447 (3)
S1A	0.34859 (5)	0.27202 (12)	0.64777 (3)	0.0152 (2)	0.447 (3)
C1B	0.4338 (4)	0.1902 (9)	0.49086 (17)	0.0330 (19)	0.553 (3)
C2B	0.4878 (3)	0.1764 (9)	0.5353 (2)	0.039 (2)	0.553 (3)
H2B	0.5421	0.1482	0.5330	0.047*	0.553 (3)
C3B	0.4624 (3)	0.2038 (10)	0.58303 (17)	0.033 (2)	0.553 (3)
H3B	0.4993	0.1943	0.6134	0.039*	0.553 (3)
C4B	0.3829 (4)	0.2450 (13)	0.5863 (2)	0.016 (3)	0.553 (3)
C5B	0.3289 (3)	0.2588 (15)	0.5419 (3)	0.0163 (8)	0.55
H5B	0.2746	0.2870	0.5442	0.020*	0.553 (3)
C6B	0.3543 (3)	0.2314 (13)	0.4942 (2)	0.027 (4)	0.553 (3)
H6B	0.3174	0.2409	0.4638	0.033*	0.553 (3)
C7B	0.4610 (6)	0.1629 (15)	0.4382 (3)	0.046 (2)	0.553 (3)
H7BA	0.4996	0.0651	0.4399	0.069*	0.553 (3)
H7BB	0.4863	0.2717	0.4277	0.069*	0.553 (3)
H7BC	0.4148	0.1342	0.4130	0.069*	0.553 (3)
O1B	0.41953 (15)	0.2857 (4)	0.68544 (10)	0.0208 (6)	0.55
O2B	0.29813 (16)	0.4298 (3)	0.64542 (10)	0.0208 (6)	0.55
O3B	0.30363 (16)	0.1087 (3)	0.65189 (10)	0.0208 (6)	0.55
S1B	0.34859 (5)	0.27202 (12)	0.64777 (3)	0.0152 (2)	0.55
C41	0.1861 (2)	0.9052 (5)	0.48107 (14)	0.0197 (8)	
H41A	0.2323	0.9814	0.4935	0.024*	
H41B	0.1387	0.9830	0.4741	0.024*	
C42	0.2016 (3)	0.8186 (6)	0.43110 (15)	0.0294 (10)	
H42A	0.2035	0.9099	0.4047	0.044*	
H42B	0.1586	0.7344	0.4200	0.044*	
H42C	0.2528	0.7555	0.4363	0.044*	
C43	0.0994 (2)	0.6613 (5)	0.50777 (14)	0.0198 (8)	
H43A	0.0908	0.5841	0.5371	0.024*	
H43B	0.1105	0.5834	0.4791	0.024*	
C44	0.0231 (2)	0.7642 (5)	0.49120 (15)	0.0233 (8)	
H44A	0.0298	0.8362	0.4609	0.035*	
H44B	0.0115	0.8419	0.5193	0.035*	
H44C	−0.0212	0.6810	0.4827	0.035*	
C45	0.2424 (2)	0.6505 (5)	0.53511 (14)	0.0196 (8)	
H45A	0.2318	0.5717	0.5637	0.024*	
H45B	0.2457	0.5749	0.5046	0.024*	
C46	0.3226 (2)	0.7396 (6)	0.54957 (17)	0.0284 (9)	
H46A	0.3361	0.8096	0.5204	0.043*	
H46B	0.3636	0.6490	0.5585	0.043*	
H46C	0.3197	0.8178	0.5791	0.043*	
C47	0.1613 (2)	0.8873 (5)	0.57032 (13)	0.0175 (8)	
H47A	0.2102	0.9586	0.5798	0.021*	
H47B	0.1166	0.9710	0.5610	0.021*	
C48	0.1444 (2)	0.7823 (5)	0.61699 (14)	0.0194 (8)	
H48A	0.1860	0.6922	0.6250	0.029*	
H48B	0.0922	0.7243	0.6098	0.029*	
H48C	0.1441	0.8626	0.6463	0.029*	
N41	0.17227 (17)	0.7763 (4)	0.52337 (11)	0.0143 (6)	
C31	0.4385 (2)	0.8762 (5)	0.72905 (13)	0.0171 (8)	
H31A	0.4868	0.9464	0.7413	0.021*	
H31B	0.3936	0.9605	0.7214	0.021*	
C32	0.4520 (3)	0.7833 (6)	0.67967 (15)	0.0260 (9)	
H32A	0.5004	0.7105	0.6857	0.039*	
H32B	0.4583	0.8720	0.6532	0.039*	
H32C	0.4061	0.7074	0.6682	0.039*	
C33	0.3440 (2)	0.6490 (5)	0.75647 (13)	0.0148 (7)	
H33A	0.3527	0.5692	0.7277	0.018*	
H33B	0.3333	0.5737	0.7858	0.018*	
C34	0.2712 (2)	0.7617 (5)	0.74017 (14)	0.0178 (8)	
H34A	0.2599	0.8362	0.7690	0.027*	
H34B	0.2252	0.6848	0.7296	0.027*	
H34C	0.2811	0.8374	0.7113	0.027*	
C35	0.4117 (2)	0.8715 (5)	0.81795 (13)	0.0174 (8)	
H35A	0.4618	0.9397	0.8264	0.021*	
H35B	0.3684	0.9580	0.8080	0.021*	
C36	0.3941 (2)	0.7765 (5)	0.86586 (14)	0.0196 (8)	
H36A	0.3916	0.8629	0.8935	0.029*	
H36B	0.4364	0.6901	0.8763	0.029*	
H36C	0.3427	0.7149	0.8589	0.029*	
C37	0.4863 (2)	0.6190 (5)	0.78468 (15)	0.0181 (8)	
H37A	0.4884	0.5427	0.7542	0.022*	
H37B	0.4723	0.5424	0.8129	0.022*	
C38	0.5688 (2)	0.6967 (6)	0.80046 (17)	0.0284 (9)	
H38A	0.5670	0.7768	0.8297	0.043*	
H38B	0.5858	0.7628	0.7716	0.043*	
H38C	0.6068	0.6006	0.8103	0.043*	
N31	0.42024 (17)	0.7550 (4)	0.77214 (11)	0.0140 (6)	
C21	0.1825 (2)	0.3831 (5)	0.24019 (15)	0.0222 (8)	
C22	0.1070 (2)	0.3169 (5)	0.24493 (14)	0.0173 (8)	
H22	0.0717	0.2895	0.2148	0.021*	
C23	0.0824 (2)	0.2902 (5)	0.29249 (14)	0.0156 (7)	
H23	0.0303	0.2460	0.2947	0.019*	
C24	0.1334 (2)	0.3274 (4)	0.33731 (14)	0.0140 (7)	
C25	0.2094 (2)	0.3924 (5)	0.33320 (15)	0.0204 (8)	
H25	0.2450	0.4174	0.3633	0.025*	
C26	0.2332 (2)	0.4210 (6)	0.28522 (16)	0.0264 (9)	
H26	0.2849	0.4672	0.2829	0.032*	
C27	0.2089 (3)	0.4133 (6)	0.18794 (16)	0.0331 (10)	
H27A	0.1626	0.4449	0.1633	0.050*	
H27B	0.2333	0.3046	0.1766	0.050*	
H27C	0.2481	0.5100	0.1901	0.050*	
O21	0.05965 (16)	0.1235 (3)	0.39491 (10)	0.0215 (6)	
O22	0.04246 (15)	0.4433 (3)	0.40172 (9)	0.0194 (6)	
O23	0.16880 (16)	0.3076 (4)	0.43637 (10)	0.0235 (6)	
S21	0.09844 (5)	0.29743 (12)	0.39789 (3)	0.0154 (2)	

### Atomic displacement parameters (Å^2^)

**Table:**

	*U*^11^	*U*^22^	*U*^33^	*U*^12^	*U*^13^	*U*^23^
C1A	0.019 (4)	0.016 (4)	0.019 (4)	0.009 (4)	0.005 (3)	0.004 (4)
C2A	0.015 (4)	0.033 (5)	0.021 (4)	0.002 (4)	0.005 (3)	−0.003 (4)
C3A	0.016 (4)	0.010 (4)	0.027 (5)	0.004 (3)	−0.005 (3)	−0.002 (4)
C4A	0.022 (7)	0.019 (6)	0.015 (7)	−0.003 (4)	−0.003 (5)	−0.005 (4)
C5A	0.0181 (18)	0.012 (2)	0.019 (2)	−0.0004 (14)	0.0032 (15)	0.0023 (15)
C6A	0.023 (8)	0.014 (5)	0.011 (7)	0.008 (5)	−0.008 (6)	−0.001 (4)
C7A	0.030 (5)	0.051 (7)	0.018 (5)	0.003 (5)	0.014 (4)	0.006 (4)
O1A	0.0218 (14)	0.0225 (14)	0.0168 (13)	0.0000 (11)	−0.0033 (11)	0.0001 (11)
O2A	0.0254 (14)	0.0151 (13)	0.0217 (14)	0.0038 (11)	0.0027 (11)	0.0016 (11)
O3A	0.0260 (14)	0.0123 (13)	0.0239 (14)	−0.0019 (11)	0.0020 (11)	−0.0002 (11)
S1A	0.0177 (5)	0.0139 (4)	0.0137 (5)	0.0012 (3)	0.0005 (3)	0.0003 (3)
C1B	0.038 (5)	0.035 (5)	0.027 (4)	−0.012 (4)	0.009 (3)	−0.008 (4)
C2B	0.025 (4)	0.060 (6)	0.034 (5)	−0.002 (4)	0.006 (3)	−0.005 (4)
C3B	0.018 (4)	0.059 (6)	0.020 (4)	0.002 (4)	0.000 (3)	0.003 (4)
C4B	0.019 (6)	0.010 (4)	0.019 (6)	0.000 (3)	0.003 (4)	0.008 (3)
C5B	0.0181 (18)	0.012 (2)	0.019 (2)	−0.0004 (14)	0.0032 (15)	0.0023 (15)
C6B	0.034 (8)	0.024 (5)	0.025 (8)	−0.013 (5)	0.008 (6)	0.000 (4)
C7B	0.042 (5)	0.070 (7)	0.029 (5)	−0.009 (5)	0.012 (4)	−0.009 (5)
O1B	0.0218 (14)	0.0225 (14)	0.0168 (13)	0.0000 (11)	−0.0033 (11)	0.0001 (11)
O2B	0.0254 (14)	0.0151 (13)	0.0217 (14)	0.0038 (11)	0.0027 (11)	0.0016 (11)
O3B	0.0260 (14)	0.0123 (13)	0.0239 (14)	−0.0019 (11)	0.0020 (11)	−0.0002 (11)
S1B	0.0177 (5)	0.0139 (4)	0.0137 (5)	0.0012 (3)	0.0005 (3)	0.0003 (3)
C41	0.0232 (19)	0.0195 (19)	0.0164 (19)	0.0010 (16)	0.0024 (15)	0.0068 (15)
C42	0.039 (2)	0.032 (2)	0.019 (2)	0.0084 (19)	0.0101 (18)	0.0072 (17)
C43	0.0232 (19)	0.0184 (19)	0.0166 (19)	−0.0037 (16)	−0.0019 (15)	−0.0031 (15)
C44	0.0212 (19)	0.027 (2)	0.020 (2)	−0.0043 (16)	−0.0015 (15)	−0.0027 (16)
C45	0.0230 (19)	0.0193 (19)	0.0165 (19)	0.0055 (16)	0.0029 (15)	0.0014 (15)
C46	0.019 (2)	0.030 (2)	0.036 (2)	0.0045 (17)	−0.0011 (17)	0.0095 (19)
C47	0.0221 (19)	0.0155 (18)	0.0141 (18)	0.0006 (15)	−0.0005 (14)	−0.0023 (14)
C48	0.026 (2)	0.0190 (19)	0.0135 (18)	0.0012 (16)	0.0025 (15)	−0.0004 (15)
N41	0.0164 (15)	0.0150 (15)	0.0112 (15)	0.0019 (12)	0.0000 (12)	0.0002 (12)
C31	0.0194 (18)	0.0154 (18)	0.0161 (18)	−0.0038 (14)	0.0008 (14)	0.0052 (14)
C32	0.034 (2)	0.027 (2)	0.019 (2)	−0.0003 (18)	0.0076 (17)	0.0053 (17)
C33	0.0171 (17)	0.0138 (17)	0.0131 (17)	−0.0049 (14)	0.0003 (14)	−0.0016 (14)
C34	0.0153 (17)	0.0177 (19)	0.0197 (19)	−0.0031 (14)	−0.0003 (14)	0.0022 (15)
C35	0.0247 (19)	0.0124 (17)	0.0138 (18)	−0.0006 (15)	−0.0022 (14)	−0.0035 (14)
C36	0.025 (2)	0.0194 (19)	0.0134 (18)	−0.0016 (16)	0.0002 (15)	−0.0023 (15)
C37	0.0175 (18)	0.0132 (18)	0.023 (2)	0.0022 (14)	−0.0009 (15)	0.0030 (15)
C38	0.020 (2)	0.024 (2)	0.039 (3)	−0.0002 (17)	−0.0049 (18)	0.0075 (19)
N31	0.0175 (15)	0.0100 (14)	0.0139 (15)	−0.0013 (12)	−0.0013 (12)	−0.0005 (12)
C21	0.024 (2)	0.0189 (19)	0.026 (2)	0.0070 (16)	0.0124 (16)	0.0057 (16)
C22	0.0203 (18)	0.0142 (18)	0.0168 (18)	0.0034 (15)	0.0004 (14)	−0.0006 (14)
C23	0.0152 (17)	0.0109 (17)	0.0205 (19)	−0.0004 (14)	0.0020 (14)	−0.0003 (14)
C24	0.0150 (17)	0.0085 (16)	0.0189 (18)	0.0041 (13)	0.0034 (14)	−0.0017 (13)
C25	0.0157 (18)	0.022 (2)	0.023 (2)	0.0026 (15)	−0.0009 (15)	−0.0019 (16)
C26	0.0158 (18)	0.031 (2)	0.034 (2)	−0.0025 (16)	0.0065 (16)	0.0013 (18)
C27	0.033 (2)	0.041 (3)	0.028 (2)	0.004 (2)	0.0165 (19)	0.007 (2)
O21	0.0262 (14)	0.0200 (14)	0.0192 (14)	−0.0025 (11)	0.0066 (11)	0.0029 (11)
O22	0.0215 (13)	0.0209 (14)	0.0155 (13)	0.0042 (11)	0.0016 (10)	−0.0017 (11)
O23	0.0229 (14)	0.0278 (15)	0.0179 (14)	0.0058 (12)	−0.0043 (11)	0.0020 (11)
S21	0.0169 (4)	0.0156 (4)	0.0132 (4)	0.0035 (3)	−0.0005 (3)	0.0005 (3)

### Geometric parameters (Å, º)

**Table:**

C1A—C2A	1.3900	C47—C48	1.515 (5)
C1A—C6A	1.3900	C47—N41	1.520 (4)
C1A—C7A	1.525 (10)	C47—H47A	0.9900
C2A—C3A	1.3900	C47—H47B	0.9900
C2A—H2A	0.9500	C48—H48A	0.9800
C3A—C4A	1.3900	C48—H48B	0.9800
C3A—H3A	0.9500	C48—H48C	0.9800
C4A—C5A	1.3900	C31—N31	1.515 (4)
C4A—S1A	1.792 (5)	C31—C32	1.516 (5)
C5A—C6A	1.3900	C31—H31A	0.9900
C5A—H5A	0.9500	C31—H31B	0.9900
C6A—H6A	0.9500	C32—H32A	0.9800
C7A—H7AA	0.9800	C32—H32B	0.9800
C7A—H7AB	0.9800	C32—H32C	0.9800
C7A—H7AC	0.9800	C33—C34	1.511 (5)
O1A—S1A	1.456 (3)	C33—N31	1.526 (4)
O2A—S1A	1.460 (3)	C33—H33A	0.9900
O3A—S1A	1.457 (3)	C33—H33B	0.9900
C1B—C2B	1.3900	C34—H34A	0.9800
C1B—C6B	1.3900	C34—H34B	0.9800
C1B—C7B	1.526 (9)	C34—H34C	0.9800
C2B—C3B	1.3900	C35—C36	1.509 (5)
C2B—H2B	0.9500	C35—N31	1.510 (4)
C3B—C4B	1.3900	C35—H35A	0.9900
C3B—H3B	0.9500	C35—H35B	0.9900
C4B—C5B	1.3900	C36—H36A	0.9800
C5B—C6B	1.3900	C36—H36B	0.9800
C5B—H5B	0.9500	C36—H36C	0.9800
C6B—H6B	0.9500	C37—N31	1.519 (4)
C7B—H7BA	0.9800	C37—C38	1.518 (5)
C7B—H7BB	0.9800	C37—H37A	0.9900
C7B—H7BC	0.9800	C37—H37B	0.9900
C41—N41	1.516 (4)	C38—H38A	0.9800
C41—C42	1.517 (5)	C38—H38B	0.9800
C41—H41A	0.9900	C38—H38C	0.9800
C41—H41B	0.9900	C21—C22	1.389 (5)
C42—H42A	0.9800	C21—C26	1.397 (6)
C42—H42B	0.9800	C21—C27	1.512 (5)
C42—H42C	0.9800	C22—C23	1.379 (5)
C43—N41	1.517 (5)	C22—H22	0.9500
C43—C44	1.519 (5)	C23—C24	1.395 (5)
C43—H43A	0.9900	C23—H23	0.9500
C43—H43B	0.9900	C24—C25	1.389 (5)
C44—H44A	0.9800	C24—S21	1.779 (4)
C44—H44B	0.9800	C25—C26	1.386 (6)
C44—H44C	0.9800	C25—H25	0.9500
C45—C46	1.515 (5)	C26—H26	0.9500
C45—N41	1.517 (4)	C27—H27A	0.9800
C45—H45A	0.9900	C27—H27B	0.9800
C45—H45B	0.9900	C27—H27C	0.9800
C46—H46A	0.9800	O21—S21	1.463 (3)
C46—H46B	0.9800	O22—S21	1.461 (3)
C46—H46C	0.9800	O23—S21	1.460 (3)
			
C2A—C1A—C6A	120.0	H48A—C48—H48C	109.5
C2A—C1A—C7A	120.3 (6)	H48B—C48—H48C	109.5
C6A—C1A—C7A	119.7 (6)	C41—N41—C43	111.3 (3)
C3A—C2A—C1A	120.0	C41—N41—C45	111.1 (3)
C3A—C2A—H2A	120.0	C43—N41—C45	106.4 (3)
C1A—C2A—H2A	120.0	C41—N41—C47	106.7 (3)
C2A—C3A—C4A	120.0	C43—N41—C47	110.8 (3)
C2A—C3A—H3A	120.0	C45—N41—C47	110.6 (3)
C4A—C3A—H3A	120.0	N31—C31—C32	115.2 (3)
C5A—C4A—C3A	120.0	N31—C31—H31A	108.5
C5A—C4A—S1A	117.0 (5)	C32—C31—H31A	108.5
C3A—C4A—S1A	122.9 (5)	N31—C31—H31B	108.5
C4A—C5A—C6A	120.0	C32—C31—H31B	108.5
C4A—C5A—H5A	120.0	H31A—C31—H31B	107.5
C6A—C5A—H5A	120.0	C31—C32—H32A	109.5
C5A—C6A—C1A	120.0	C31—C32—H32B	109.5
C5A—C6A—H6A	120.0	H32A—C32—H32B	109.5
C1A—C6A—H6A	120.0	C31—C32—H32C	109.5
O1A—S1A—O3A	113.61 (16)	H32A—C32—H32C	109.5
O1A—S1A—O2A	113.33 (16)	H32B—C32—H32C	109.5
O3A—S1A—O2A	112.64 (15)	C34—C33—N31	114.2 (3)
O1A—S1A—C4A	105.4 (3)	C34—C33—H33A	108.7
O3A—S1A—C4A	109.7 (4)	N31—C33—H33A	108.7
O2A—S1A—C4A	101.1 (4)	C34—C33—H33B	108.7
C2B—C1B—C6B	120.0	N31—C33—H33B	108.7
C2B—C1B—C7B	120.6 (5)	H33A—C33—H33B	107.6
C6B—C1B—C7B	119.4 (5)	C33—C34—H34A	109.5
C3B—C2B—C1B	120.0	C33—C34—H34B	109.5
C3B—C2B—H2B	120.0	H34A—C34—H34B	109.5
C1B—C2B—H2B	120.0	C33—C34—H34C	109.5
C4B—C3B—C2B	120.0	H34A—C34—H34C	109.5
C4B—C3B—H3B	120.0	H34B—C34—H34C	109.5
C2B—C3B—H3B	120.0	C36—C35—N31	115.8 (3)
C3B—C4B—C5B	120.0	C36—C35—H35A	108.3
C6B—C5B—C4B	120.0	N31—C35—H35A	108.3
C6B—C5B—H5B	120.0	C36—C35—H35B	108.3
C4B—C5B—H5B	120.0	N31—C35—H35B	108.3
C5B—C6B—C1B	120.0	H35A—C35—H35B	107.4
C5B—C6B—H6B	120.0	C35—C36—H36A	109.5
C1B—C6B—H6B	120.0	C35—C36—H36B	109.5
C1B—C7B—H7BA	109.5	H36A—C36—H36B	109.5
C1B—C7B—H7BB	109.5	C35—C36—H36C	109.5
H7BA—C7B—H7BB	109.5	H36A—C36—H36C	109.5
C1B—C7B—H7BC	109.5	H36B—C36—H36C	109.5
H7BA—C7B—H7BC	109.5	N31—C37—C38	114.9 (3)
H7BB—C7B—H7BC	109.5	N31—C37—H37A	108.5
N41—C41—C42	114.6 (3)	C38—C37—H37A	108.5
N41—C41—H41A	108.6	N31—C37—H37B	108.5
C42—C41—H41A	108.6	C38—C37—H37B	108.5
N41—C41—H41B	108.6	H37A—C37—H37B	107.5
C42—C41—H41B	108.6	C37—C38—H38A	109.5
H41A—C41—H41B	107.6	C37—C38—H38B	109.5
C41—C42—H42A	109.5	H38A—C38—H38B	109.5
C41—C42—H42B	109.5	C37—C38—H38C	109.5
H42A—C42—H42B	109.5	H38A—C38—H38C	109.5
C41—C42—H42C	109.5	H38B—C38—H38C	109.5
H42A—C42—H42C	109.5	C35—N31—C31	107.0 (3)
H42B—C42—H42C	109.5	C35—N31—C37	111.2 (3)
N41—C43—C44	114.4 (3)	C31—N31—C37	110.8 (3)
N41—C43—H43A	108.6	C35—N31—C33	110.9 (3)
C44—C43—H43A	108.6	C31—N31—C33	111.0 (3)
N41—C43—H43B	108.6	C37—N31—C33	106.0 (3)
C44—C43—H43B	108.6	C22—C21—C26	117.9 (3)
H43A—C43—H43B	107.6	C22—C21—C27	121.0 (4)
C43—C44—H44A	109.5	C26—C21—C27	121.2 (4)
C43—C44—H44B	109.5	C23—C22—C21	121.2 (3)
H44A—C44—H44B	109.5	C23—C22—H22	119.4
C43—C44—H44C	109.5	C21—C22—H22	119.4
H44A—C44—H44C	109.5	C22—C23—C24	120.6 (3)
H44B—C44—H44C	109.5	C22—C23—H23	119.7
C46—C45—N41	115.0 (3)	C24—C23—H23	119.7
C46—C45—H45A	108.5	C25—C24—C23	118.8 (3)
N41—C45—H45A	108.5	C25—C24—S21	121.9 (3)
C46—C45—H45B	108.5	C23—C24—S21	119.3 (3)
N41—C45—H45B	108.5	C26—C25—C24	120.1 (3)
H45A—C45—H45B	107.5	C26—C25—H25	119.9
C45—C46—H46A	109.5	C24—C25—H25	119.9
C45—C46—H46B	109.5	C25—C26—C21	121.3 (4)
H46A—C46—H46B	109.5	C25—C26—H26	119.3
C45—C46—H46C	109.5	C21—C26—H26	119.3
H46A—C46—H46C	109.5	C21—C27—H27A	109.5
H46B—C46—H46C	109.5	C21—C27—H27B	109.5
C48—C47—N41	115.0 (3)	H27A—C27—H27B	109.5
C48—C47—H47A	108.5	C21—C27—H27C	109.5
N41—C47—H47A	108.5	H27A—C27—H27C	109.5
C48—C47—H47B	108.5	H27B—C27—H27C	109.5
N41—C47—H47B	108.5	O23—S21—O22	112.92 (16)
H47A—C47—H47B	107.5	O23—S21—O21	113.78 (16)
C47—C48—H48A	109.5	O22—S21—O21	112.88 (16)
C47—C48—H48B	109.5	O23—S21—C24	106.16 (16)
H48A—C48—H48B	109.5	O22—S21—C24	104.88 (15)
C47—C48—H48C	109.5	O21—S21—C24	105.23 (16)

### Hydrogen-bond geometry (Å, º)

**Table:**

*D*—H···*A*	*D*—H	H···*A*	*D*···*A*	*D*—H···*A*
C6*B*—H6*B*···O23	0.95	2.57	3.351 (6)	140
C31—H31*B*···O3*B*^i^	0.99	2.49	3.344 (4)	145
C33—H33*A*···O2*B*	0.99	2.47	3.354 (4)	148
C35—H35*A*···O22^ii^	0.99	2.42	3.228 (4)	138
C36—H36*C*···O3*B*^iii^	0.98	2.58	3.544 (4)	169
C43—H43*B*···O22	0.99	2.44	3.269 (4)	141
C45—H45*A*···O2*B*	0.99	2.53	3.367 (4)	142
C47—H47*A*···O3*B*^i^	0.99	2.57	3.440 (4)	147
C48—H48*B*···O22^iv^	0.98	2.58	3.562 (4)	175

Symmetry codes: (i) *x*, *y*+1, *z*; (ii) *x*+1/2, −*y*+3/2, *z*+1/2; (iii) −*x*+1/2, *y*+1/2, −*z*+3/2; (iv) −*x*, −*y*+1, −*z*+1.
